# *Xa7*, a Small Orphan Gene Harboring Promoter Trap for AvrXa7, Leads to the Durable Resistance to *Xanthomonas oryzae* Pv. *oryzae*

**DOI:** 10.1186/s12284-021-00490-z

**Published:** 2021-05-30

**Authors:** Congying Wang, Shen Chen, Aiqing Feng, Jing Su, Wenjuan Wang, Jinqi Feng, Bing Chen, Meiying Zhang, Jianyuan Yang, Liexian Zeng, Xiaoyuan Zhu

**Affiliations:** Guangdong Provincial Key Laboratory of High Technology for Plant Protection, Plant Protection Research Institute, Guangdong Academy of Agricultural Sciences, Guangzhou, 510640 China

**Keywords:** *Xa7*, Map-based cloning, Rice bacterial blight, Durable resistance, TALE, *Xanthomonas*, Executor gene, Orphan gene, Gene origin, Genomic structural variation

## Abstract

**Background:**

The rice (*Oryza sativa*) gene *Xa7* has been hypothesized to be a typical executor resistance gene against *Xanthomonas oryzae* pv. *oryzae* (*Xoo*), and has conferred durable resistance in the field for decades. Its identity and the molecular mechanisms underlying this resistance remain elusive.

**Results:**

Here, we filled in gaps of genome in *Xa7* mapping locus via BAC library construction, revealing the presence of a 100-kb non-collinear sequence in the line IRBB7 compared with Nipponbare reference genomes. Complementary transformation with sequentially overlapping subclones of the BACs demonstrated that *Xa7* is an orphan gene, encoding a small novel protein distinct from any other resistance proteins reported. A 27-bp effector binding element (EBE) in the *Xa7* promoter is essential for AvrXa7-inducing expression model. XA7 is anchored in the endoplasmic reticulum membrane and triggers programmed cell death in rice and tobacco (*Nicotiana benthamiana*). The *Xa7* gene is absent in most cultivars, landraces, and wild rice accessions, but highly homologs of XA7 were identified in *Leersia perrieri*, the nearest outgroup of the genus *Oryza*.

**Conclusions:**

*Xa7* acts as a trap to perceive AvrXa7 via EBE_AvrXa7_ in its promoter, leading to the initiation of resistant reaction. Since EBE_AvrXa7_ is ubiquitous in promoter of rice susceptible gene *SWEET14*, the elevated expression of which is conducive to the proliferation of *Xoo*, that lends a great benefit for the *Xoo* strains retaining AvrXa7. As a result, varieties harboring *Xa7* would show more durable resistance in the field. *Xa7* alleles analysis suggests that the discovery of new resistance genes could be extended beyond wild rice, to include wild grasses such as *Leersia* species.

**Supplementary Information:**

The online version contains supplementary material available at 10.1186/s12284-021-00490-z.

## Introduction

Deciphering the origin and evolution of plant resistance (*R*) genes accelerates understanding the mechanisms of plant durable disease resistance, of which can enable the sustainable management of crop diseases through the utilization of host resistance. The general routes by which new genes originate include exon shuffling, gene duplication and subsequent divergence, retroposition, the transposition of mobile elements, lateral gene transfer, gene fusion and fission, and de novo origination (Long et al. 2003). De novo originated genes in a genome are usually present in the form of orphan genes (Khalturin et al. 2009). Orphan genes (or taxonomically restricted genes) are phylogenetically restricted, without detectable sequence similarity in the genomes of other organisms and do not encode any previously identified protein domains (Khalturin et al. 2009). Orphan genes generally have no introns, encode small proteins, undergo more rapid evolution than other genes in the same genome, and more likely to be expressed under environmental pressure than non-orphan genes (Guo et al. 2007). Recent studies revealed that orphan genes are important for key agronomic traits; for example, *Ms2* confers male sterility in wheat (*Triticum aestivum*) (Ni et al. 2017); *QQS* (encoding Qua-Quine Starch) regulates carbon and nitrogen partitioning across species (Li et al. 2015); *Oryza sativa Defense-Responsive Gene 10* (*OSDR10*) negatively regulates pathogen-induced defense response in rice (Xiao et al. 2009) and *Triticum aestivum Fusarium Resistance Orphan Gene* (*TaFROG*) enhances wheat resistance to disease (Perochon et al. 2015). The lack of characterized domains makes it difficult to study their molecular mechanisms however, relatively few plant orphan genes have been studied in depth. The characterization of additional orphan genes would be helpful for examining both their biochemical modes of action and evolutionary origins.

Interactions between rice and *Xanthomonas oryzae* (*Xo*) have become important models for understanding the fundamental aspects of plant disease resistance and bacterial pathogenesis, as well as other aspects of plant and microbial biology, with implications for animal innate immunity too (Niño-Liu et al. 2006). The elucidation of the diverse molecular mechanisms involved in rice-*Xo* interactions has substantially contributed to both fundamental research and crop breeding practices (Jiang et al. 2020). So far, 17 genes (or alleles) against *Xo* have been cloned, which can be divided into six categories according to their gene structures and molecular mechanisms: (1) *Xa1*/*Xa1–2*/*Xa2*/*Xa14*/*Xa31(t)*/*Xa45*(t), encoding atypical nucleotide binding site–leucine–rich repeat (NBS–LRR) containing proteins (Yoshimura et al. 1998; Ji et al. 2020; Zhang et al. 2020); (2) *Xa3*/*Xa26* and *Xa21*, encoding extracellular LRR receptor kinases (Sun et al. 2004; Xiang et al. 2006; Song et al. 1995); (3) *Xa4*, encoding a cell wall–associated kinase (Hu et al. 2017); (4) *xa5*, encoding a small subunit of a general transcription factor (Iyer and Mccouch, 2004; Jiang et al. 2006); (5) *xa13*, *xa25* and *xa41*(t), encoding a family of sugar transporters (Yang et al. 2006; Liu et al. 2011; Hutin et al. 2016); and (6) the executor *R* genes *Xa27*, *Xa10*, and *Xa23*, (Gu et al. 2005; Tian et al. 2014; Wang et al. 2015). Most of them are involved in the pathogenesis pathway mediated by the *X*o type-III secretion system (T3SS) (Jiang et al. 2020). *Xo* T3SS transports transcription activator–like effectors (TALEs) into host plant cells to elicit disease (White and Yang, 2009). TALEs promote host gene expression by directly binding to specific sequences, termed effector binding elements (EBEs), in the target gene promoter via repeat-variable diresidues (RVDs) in the central repeat region; thus, the specificity of RVD-mediated DNA binding has been successfully decoded (Boch et al. 2009; Moscou and Bogdanove, 2009). The number and composition of RVDs in TALEs can therefore be used to computationally predict the EBEs that can be recognized by the effector, and accordingly allow the prediction of candidate target genes (Doyle et al. 2012; Grau et al. 2013). The avirulent gene AvrXa7, a typical TALE, has dual functions of avirulence and virulence (Yang et al. 2000; Vera Cruz et al. 2000). The RVDs of AvrXa7 would facilitate the rapid scanning and prediction of *Xa7* candidates.

*Xa7* is well known for conferring durable resistance against rice bacterial blight, which has been verified by 10-year cropping period (Webb et al., 2010). Furthermore, increased effectiveness has been reported for *Xa7*-mediated resistance under high temperature and drought stress (Webb et al. 2010; Dossa et al., 2020). These features lend great importance to functional study of this gene. *Xa7* was originally identified from rice variety DV85 (Sidhu et al. 1978) and introduced into the near-isogenic line IRBB7 through multiple rounds of backcrossing of DV85 with IR24 as a recurrent parent (Ogawa et al. 1991). Kaji and Ogawa (1995) mapped this gene to the 107.5 cM position of chromosome 6, reporting a recombination rate of 8% with the G1091 marker (Kaji and Ogawa, 1995). Subsequently, the fine mapping of *Xa7* was independently carried out by a few research groups. Porter et al. (2003) demonstrated that *Xa7* was tightly linked to the STS marker M5, at a genetic distance of 0.16 Mb while Zhang et al. (2009) mapped the *Xa7* locus to a region between the RM20576 and MY4 markers, comprising a physical distance of approximately 200 kb (Zhang et al. 2009). Our previous work narrowed down the *Xa7* gene to a 118.5-kb interval between the molecular markers GDSSR02 and RM20593 (Chen et al. 2008).

In the present study, we focused on the target region between markers U05 and Poz, filling the gap within the mapping region in IRBB7 by constructing and sequencing a genomic BAC library. The candidate gene *Xa7* was pinpointed in the genomic gap sequence using TALgetter. We eventually confirmed the function of *Xa7* by complementary transformation of susceptible rice with the sequential over-lapping BAC subclones. The *Xa7* allele information obtained in this study provides clues about its evolutionary origin and durability of resistance.

## Results

### The *Xa7* Locus Comprises an Approximately 100 Kb Non-collinear Sequence Compared with the Rice Nipponbare Reference Genome

**Fig. 1:**
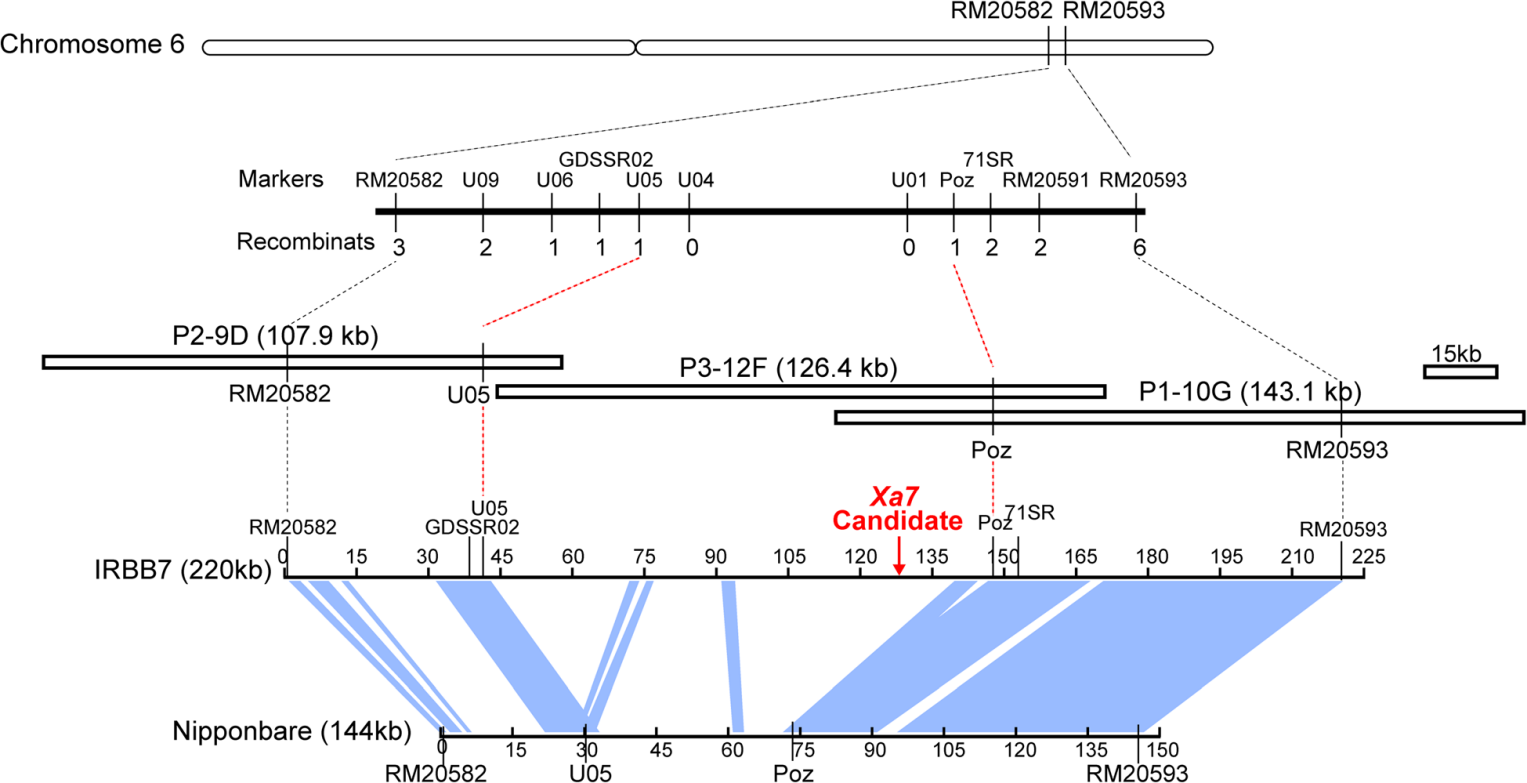
Fine mapping of *Xa7*. The vertical lines represent the sites of key markers in the physical map. The IRBB7 BAC contigs are represented by overlapping unfilled rectangles. The light blue shading indicates collinear regions of the IRBB7 vs. Nipponbare genome. The region between the red dotted lines is the mapped *Xa7* region, which is flanked by the markers U05 and Poz

We had previously mapped *Xa7* in a genetic interval of 0.28 cM between the SSR markers RM20582 and RM20593 (Chen et al. 2008). In the present study, we developed an F_2_ genetic population from a cross between an *indica* line susceptible to bacterial blight, II-32B, and the *Xa7-*containing near-isogenic line IRBB7. The recombinants were screened using the markers RM20582 and RM20593, and the key recombinants were selected to develop the F_3_ families. Homozygous individuals were selected for the mapping analysis. Polymorphic STS markers were developed between RM20582 and RM20593, including U09, U06, U05, U04, U01, and Poz (Fig. 1, Table S1). These, used in combination with the previously developed polymorphic markers GDSSR02, 71SR and RM20591, enabled us to narrow down the candidate genes to the interval between U05 and Poz. When we compared the allele sequence from IRBB7 with the reference genome of Nipponbare in this interval, we observed great variation in the genome structure. The assembly of re-sequencing data of IRBB7 released from the 3000 Rice Genome Project (The 3 k The RGP, 2014) also showed that this region has a large gap compared to the Nipponbare genome. Due to the short reads, low coverage of the sequencing and the complexity of sequence structure, the GAP region could not be filled up by re-sequencing data of IRBB7 released from 3 k RGP.

To determine the complete genomic sequence of the target region, a BAC library was constructed for IRBB7. Around 45,000 clones were generated for the library, and the sizes of their insertions ranged from 120 kb to 140 kb. The library comprised approximately 15× coverage of the whole genome. The flanking markers of U05 and Poz were used for the library screening. After multiple rounds of PCR-based screening, three positive clones were identified and isolated: P2-9D, P3-12F, and P1-10G. After enzyme digestion and electrophoresis analysis, the insertions of the positive BAC clones were estimated to be about 110 kb, 120 kb, and 150 kb, respectively. The plasmids of positive clones were sequenced using the Illumina massively parallel sequencing platform. The sequencing data was assembled to an overlapping contig of 308 kb in length, which was 102 kb longer than the corresponding region in the genome of the *japonica* rice cultivar Nipponbare (206.4 kb) and 81 kb longer than that of the *indica* rice restorer line Minghui 63 (227.5 kb). The sequence between U05 and Poz markers is 106 kb in length. The collinear analysis of the sequence corresponding to Nipponbare genome is carried out using Genomes Match (http://www.softberry.com/berry.phtml?topic=gmatch&group= programs&subgroup = scanh), revealing few collinear regions between two genomes (Fig. 1). The approximate 100-kb insertion in IRBB7 could explain why we did not obtain recombinants within the interval between U05 and Poz from the previous mapping population.

There are 35 open reading frames (ORFs) in the 106 kb interval as predicted using the Fgenesh tool (http://www.softberry.com/berry.phtml?topic=fgenesh&group= programs& subgroup = gfind). In order to pinpoint the *Xa7* candidate gene, we looked for potential AvrXa7 binding sites. The effector binding site of AvrXa7 (EBE_AvrXa7_) had been predicted using the TALgetter tool (http://galaxy2.informatik.uni-halle.de:8976) (Grau et al. 2013). Two AvrXa7 EBEs were identified with a *p*-value less than 1.0 × 10^− 6^, and only one of them was located in the promoter region of a predicted ORF and was therefore considered the candidate *Xa7* gene (Fig. 1). The candidate gene encodes a 113-aa protein. The predicted AvrXa7 EBE was located 110 ~ 136 bp upstream of the ATG (start codon) for the candidate gene.

### A Unique Small Gene Encoding a 113-Aa Protein Has Been Validated for Disease Resistance

We next tested whether the candidate gene for *Xa7* can confer *Xoo* resistance by transforming constructs carrying the locus into *Xoo*-susceptible rice varieties. The transformation-competent artificial chromosome vector (TAC) subclone library was constructed from the BAC plasmids P1-10G and P3-12F, which containing the *Xa7* candidate gene. A total of 87 positive clones were obtained by screening the subclone library with the primers used for amplifying the promoter and CDS region of the candidate gene, respectively (Table S1). The positive subclones were subjected to terminal sequencing to determine the identities of the inserts. According to the sequence information, six TAC subcloned plasmids were selected for complementary transformation into the susceptible varieties including the *indica* varieties IR24 and Zhonghua11 (ZH11) and *japonica* variety Taipei309 (TP309). The transgenic plants were inoculated with *Xanthomonas oryzae* pv. *oryzae* (*Xoo*) strain PXO86 at the booting stage to evaluate their resistance levels (Table 1).

**Table 1:** Complementary Transgenic Plants Generated in this Study

Constructs	Recipient varieties	HTP-resistant T_**0**_ plants ^a^	***Xoo***-resistant T_**0**_ plants ^b^
S1AE6	IR24	22	16
S1AG3	IR24	10	0
S2CD8	IR24	6	2
S1BA3	IR24	11	5
S1BA3	ZH11	33	11
S1BA3	TP309	15	2
CG52–1300	ZH11	14	4
CG52–1300	TP309	21	8
S2BD2	ZH11	44	13
S2BD2	TP309	39	17
S1AD4	IR24	6	0
*P*_*PR1*_:*Xa7*:*T*_*nos*_	TP309	28	21

^a^ The number of transgenic plants regenerated from hygromycin selection culture

^b^ The number of transgenic plants resistant to *Xoo* strain PXO86. To test resistance, five to ten of the uppermost fully expanded leaves of each transgenic plant were inoculated using the leaf tip-clipping method at the booting stage. The plants were scored as resistant or susceptible based on the average lesion length at two weeks after inoculation

**Fig. 2:**
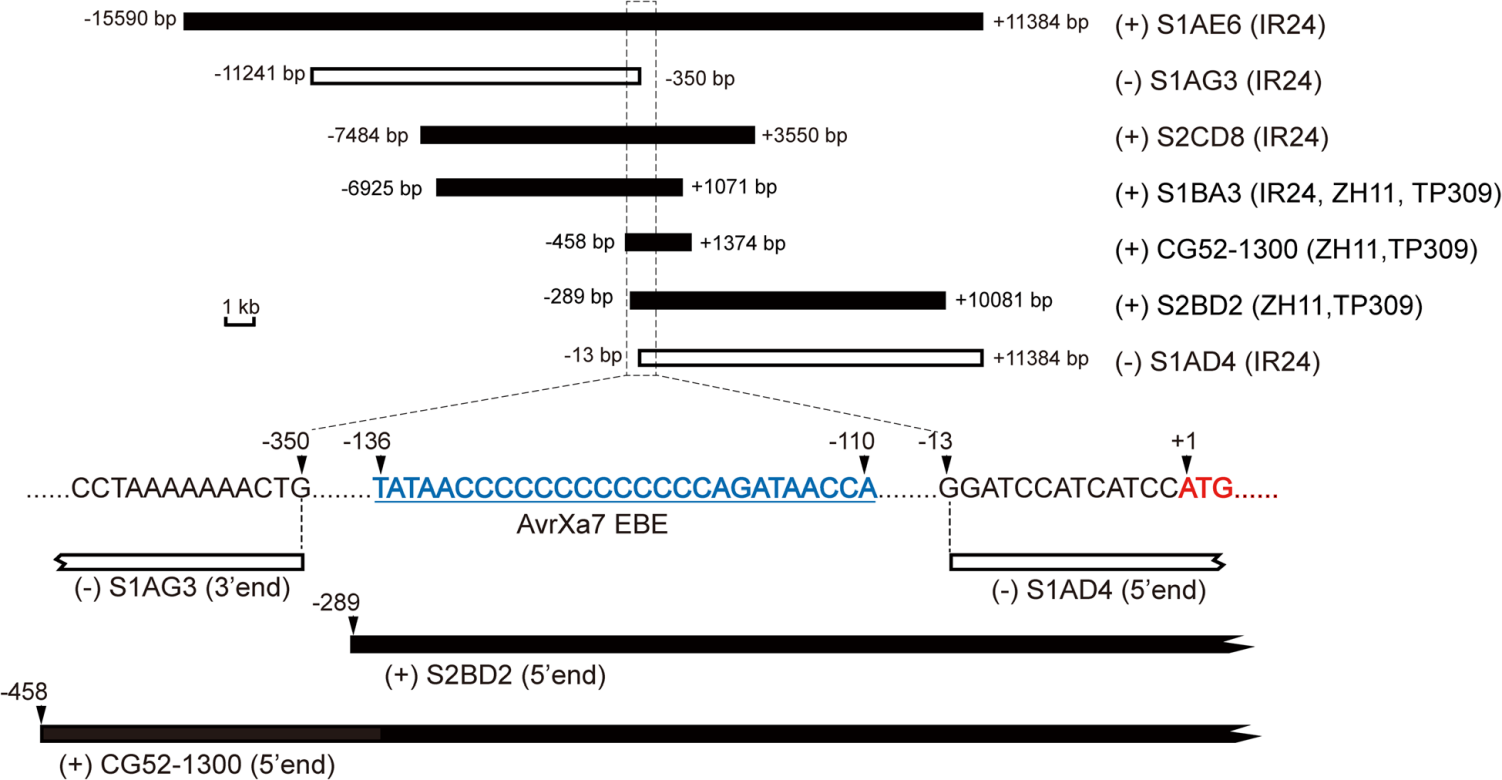
Functional complementation assay for the *Xa7* candidate gene. The TAC sub-clones used for rice transformation are indicated with empty and filled rectangles. The empty rectangles represent the subclones generating susceptible transgenic lines, while filled rectangles represent the subclones generating transgenic resistant lines. The positional information of the inserted fragments is shown flanking the rectangles. Negative numbers indicate a distance upstream of the ATG code initiation site for the candidate gene, while positive numbers indicate a downstream distance from it. The subclones that produced bacterial blight–resistant transgenic rice are marked with (+) before the clone number, otherwise they are marked with (−). The recipient varieties used for transformation are provided in brackets after the clone numbers

The positional information of the inserts from the six TAC subclones is presented in Fig. 2. The subclones S1AE6, S2CD8, S1BA3 and S2BD2 covered the full length of the promoter region and CDS region of the *Xa7* candidate gene. Among them, the 5′ end of the insert from the S2BD2 sub-clone carried only 289-bp core promoter sequence including the putative EBE_AvrXa7_. The 3′ end of the insert from the S1BA3 subclone carried only 1071 bp downstream of the translation initiation site of the candidate gene. The transgenic lines from the four subclones were all resistant to PXO86 (Table 1). On the other hand, the 3′ end of the inserted fragment from the S1AG3 subclone contained only part of the promoter sequence without the putative EBE_AvrXa7_, while the 5′ end of the inserted fragment from the S1AD4 subclone contained the full CDS region and a 13-bp untranslated region (UTR) without the putative EBE_AvrXa7_. Transgenic rice lines from both subclones were susceptible to the PXO86 (Table 1).

Subsequently, a single gene fragment containing the 458-bp promoter and full CDS sequence of *Xa7* were isolated from the IRBB7 genome using PCR-amplification and ligated into the pCambia1300 expression vector, which was named as CG52–1300. The transgenic lines integrated with this single gene fragment showed disease resistance (Table 1), therefore, this candidate gene is responsible for the resistance to the *Xoo* strain PXO86. Both of the EBE_AvrXa7_ in the promoter region and the entire CDS are indispensable for pathogen resistance (Fig. 2).

### *Xa7* Confers Resistance in Response to Pathogen Infection

**Fig. 3:**
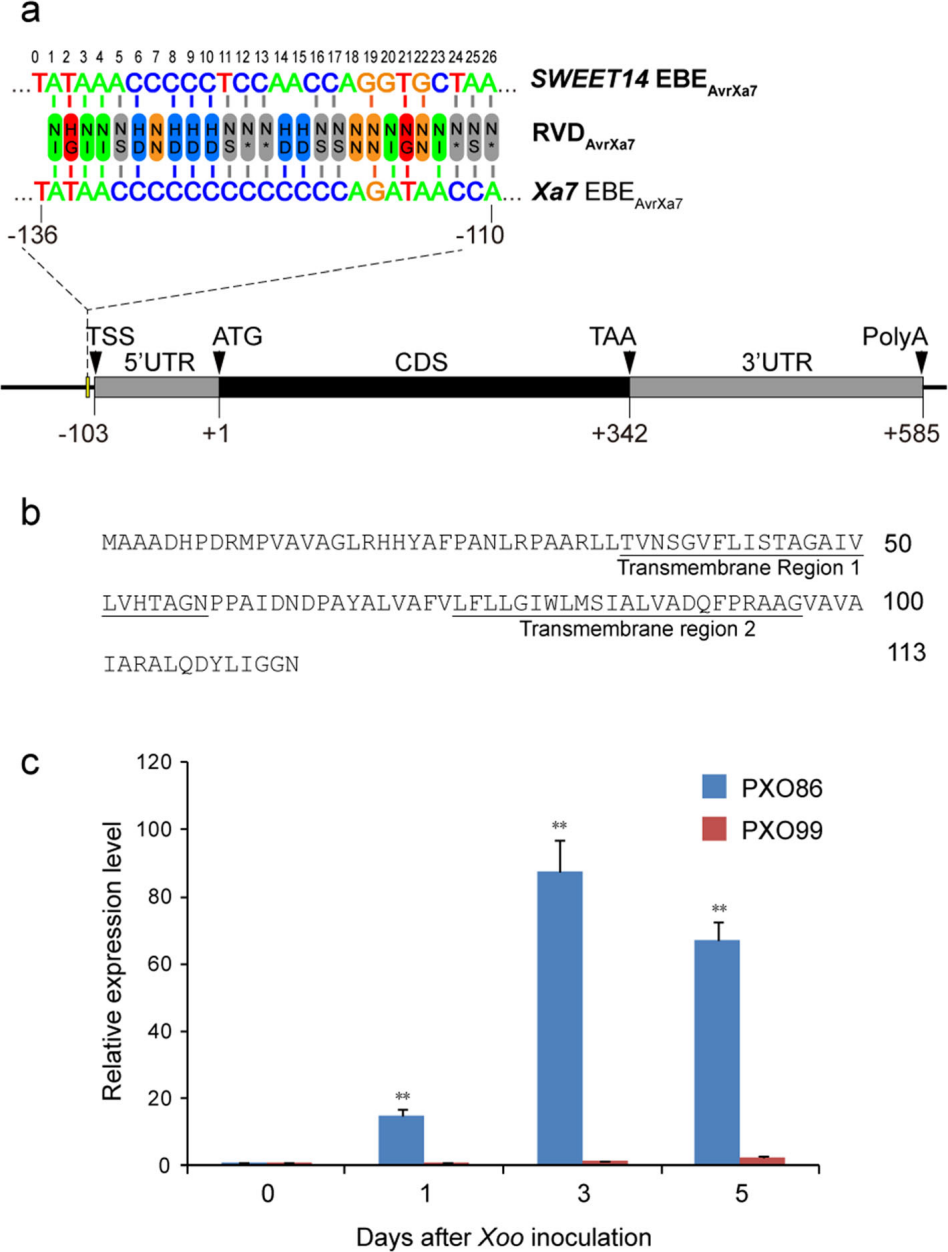
The structure and expression characterization of *Xa7*. (**a**) Gene structure of *Xa7*. The schematic map shows the EBE_AvrXa7_ (110–136 bp upstream of the ATG start code), the coding region (black rectangle), and the 5′ and 3′UTR regions (gray rectangles). The numbers indicate the distance from ATG code initiation site for each element or substructure. (**b**) The deduced amino acid sequence of XA7. The transmembrane helices are underlined. (**c**) Expression pattern of *Xa7* gene after inoculation with *Xanthomonas oryzae* pv. *oryzae* (*Xoo*) strain PXO86 and PXO99, respectively. The average expression level of *Xa7* in IRBB7 at 0 d was set as “1”. The expression levels of other time point were used to compare with that of 0d. The data represents the mean ± standard deviation (*n* = 3 replicates). Student’s *t*-test analysis indicates a significant difference (compared with control, ** *P* < 0.01)

We obtained the complete transcript sequence of *Xa7* using a RACE assay. The transcription initiation site (TSS) of *Xa7* was located at 103 bp upstream of the initiation codon (ATG). The 3′ UTR of *Xa7* is 253 bp in length, and the full transcript is 688 bp long (Fig. 3a). The *Xa7* CDS region is intronless and is predicted to encode a small protein of 113 aa in length. XA7 is an orphan protein that does not match any similar structures in pfam database. Compared with other rice executor proteins, XA27 only shared a 28.81% similarity. Its similarities with XA23 and XA10 were 12.17% and 10.85%, respectively (Fig. S2). A SOSUI program prediction (http://harrier.nagahama-i-bio.ac.jp/sosui/sosui_submit.html) (Hirokawa, et al. 1998) shows that XA7 is a membrane protein with two transmembrane helices (Fig. 3b). While XA27 and XA23 have three transmembrane helices, XA10 has four transmembrane helices. A prediction using Iupred2 (short) prediction (https://iupred2a.elte.hu/) (Erdős G, Dosztányi Z., 2020) showed that the overall score of the proteins was below 0.5 (Fig. S1). Residues with a score above 0.5 can be regarded as disordered (Dosztányi et al. 2005). The degree of intrinsic structural disorder is relevant to the “age” of a gene, as young genes are highly disordered (Wilson et al. 2017). This suggests *Xa7* is an old gene.

As shown in Fig. 3c, the expression level of *Xa7* was low before *Xoo* inoculation. Its expression began to increase 1 day after inoculation with the *Xoo* strain harboring AvrXa7 and reached a peak after 3 days; therefore, the expression of *Xa7* gene is induced by AvrXa7.

### The Promoter-Located AvrXa7 Recognition Sequence Is the Key Regulatory Element for the Pathogen-Induced Expression of *Xa7*

To further validate the function of the AvrXa7 EBE within the promoter and CDS region, we used a CRISPR/Cas9 system to generate site-specific knockout mutations in IRBB7.

**Fig. 4:**
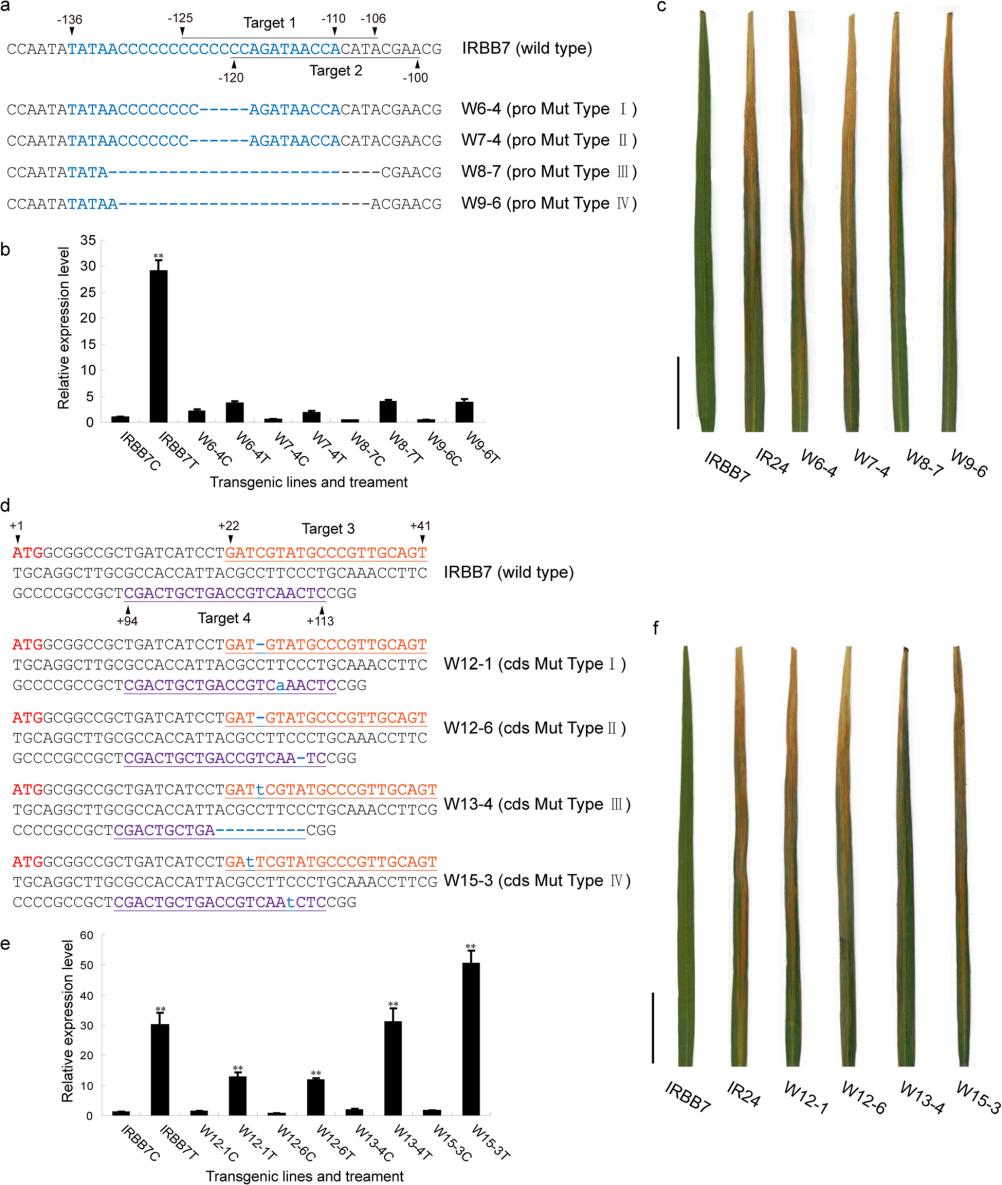
Verification of the key functional elements of the *Xa7* gene. (**a**) The key sequences in the promoter targeted by genome editing. EBE_AvrXa7_ are displayed in blue. The sgRNA targeting sequences are underlined. W6–4 and W7–4 are T_1_ homozygous transgenic lines obtained using Target1 site-specific editing, W8–7 and W9–6 are T_2_ homozygous transgenic lines obtained using Target2 site-specific editing. (**b**) Transcription of *Xa7* in the homozygous promoter mutant lines in response to *Xoo* strain PXO86. IRBB7C, W6–4C, W7–4C, W8–7C, and W9–6C stand for the lines that were inoculated with *Xoo* strain PXO86 at day0. IRBB7T, W6–4T, W7–4T, W8–7T, and W9–6T stand for the same lines inoculated with *Xoo* strain PXO86 at 3 days after inoculation. The average expression level of *Xa7* in IRBB7 at 0 d (IRBB7C) was set as “1”. The expression levels of other lines were used to compare with that of IRBB7C. “**” indicates a significant difference between IRBB7C and other plants at *P* < 0.01. (**c**) Disease phenotype of the *Xa7* homozygous promoter mutants after inoculation with the *Xoo* strain PXO86. Scale bar stands for 5 cm. (**d**) *Xa7* coding region for genome editing. The sgRNA targeting sequences are underlined. ATG code initiation site is marked in red, base insertions are marked in blue lowercase letters, while base deletions are marked as “-”. W12–1, W12–6, W13–4, and W15–3 are T_1_ homozygous transgenic lines obtained using Target3 and Target4 site-specific editing. (**e**) Transcription of *Xa7* in the homozygous CDS mutant lines in response to *Xoo* strain PXO86. IRBB7C, W12–1C, W12–6C, W13–4C, and W15–3C stand for the lines that were inoculated with *Xoo* strain PXO86 at day0. IRBB7T, W12–1 T, W12–6 T, W13–4 T, and W15–3 T stand for the same lines inoculated with *Xoo* strain PXO86 at 3 days after inoculation. The average expression level of *Xa7* in IRBB7 at 0 d (IRBB7C) was set as “1”. The expression levels of other lines were used to compare with that of IRBB7C. “**” stand for a significant difference between IRBB7C and other plants at *P* < 0.01. (**f**) Disease phenotype of *Xa7* homozygous CDS mutant lines after inoculation with the *Xoo* strain PXO86. Scale bar stands for 5 cm. Each data in (**b**) and (**e**) represents the mean ± standard deviation (*n* = 3 replicates). Student’s *t*-test analysis indicates a significant difference (compared with control, ** *P* < 0.01)

As shown in Fig. 4a, the CRISPR constructs Target1 and Target2 were designed to target the EBE_AvrXa7_ in the *Xa7* promoter region, and produced a variety of base pair deletions. Homozygous mutant lines were obtained from the T_1_ generation, and the mutation types are shown in Fig. 4a. These mutants were subsequently challenged with PXO86. The transcription of *Xa7* could no longer be activated by PXO86 in the mutants, which concomitantly lost their resistance to the pathogen (Fig. 4b, c). These results indicate that the EBE_AvrXa7_ is an essential *cis*-acting regulatory element for the transcriptional activation of *Xa7*. In previous studies, AvrXa7 has been shown to target the rice major susceptibility gene *SWEET14* for bacterial virulence (Antony et al., 2010; Hutin et al. 2016). Both *SWEET14* and *Xa7* genes carry EBE_AvrXa7_ in their promoter regions. The EBE_AvrXa7_ of *Xa7* is a close mimic of the EBE_AvrXa7_ of *SWEET14* for TALE-induced disease susceptibility (Luo et al. 2021). We compared the sequence of the EBE _AvrXa7_ in *Xa7* and that in *SWEET14*, as shown in Fig. 3a, and found that there are 9 bases that differ near the 3-’ end of the EBEs.

Similarly, the CRISPR constructs Target3 and Target4 were used to target the *Xa7* CDS region and produced different types of base pair deletions and insertions, respectively (Fig. 4d). The mutations resulted in early termination, frame shifting, and substitution mutation of the XA7 protein. Although the transcription of *Xa7* could still be activated by the pathogen, the homozygous mutation lines lost their resistances to the pathogen (Fig. 4e, f). These results suggest that the protein encoded by the *Xa7* gene is a necessary factor for performing resistance response.

### XA7, Anchored in the Endoplasmic Reticulum Membrane, Can Induce Hypersensitive Response Reactions in Mono- and Dicotyledonous Plants

**Fig. 5:**
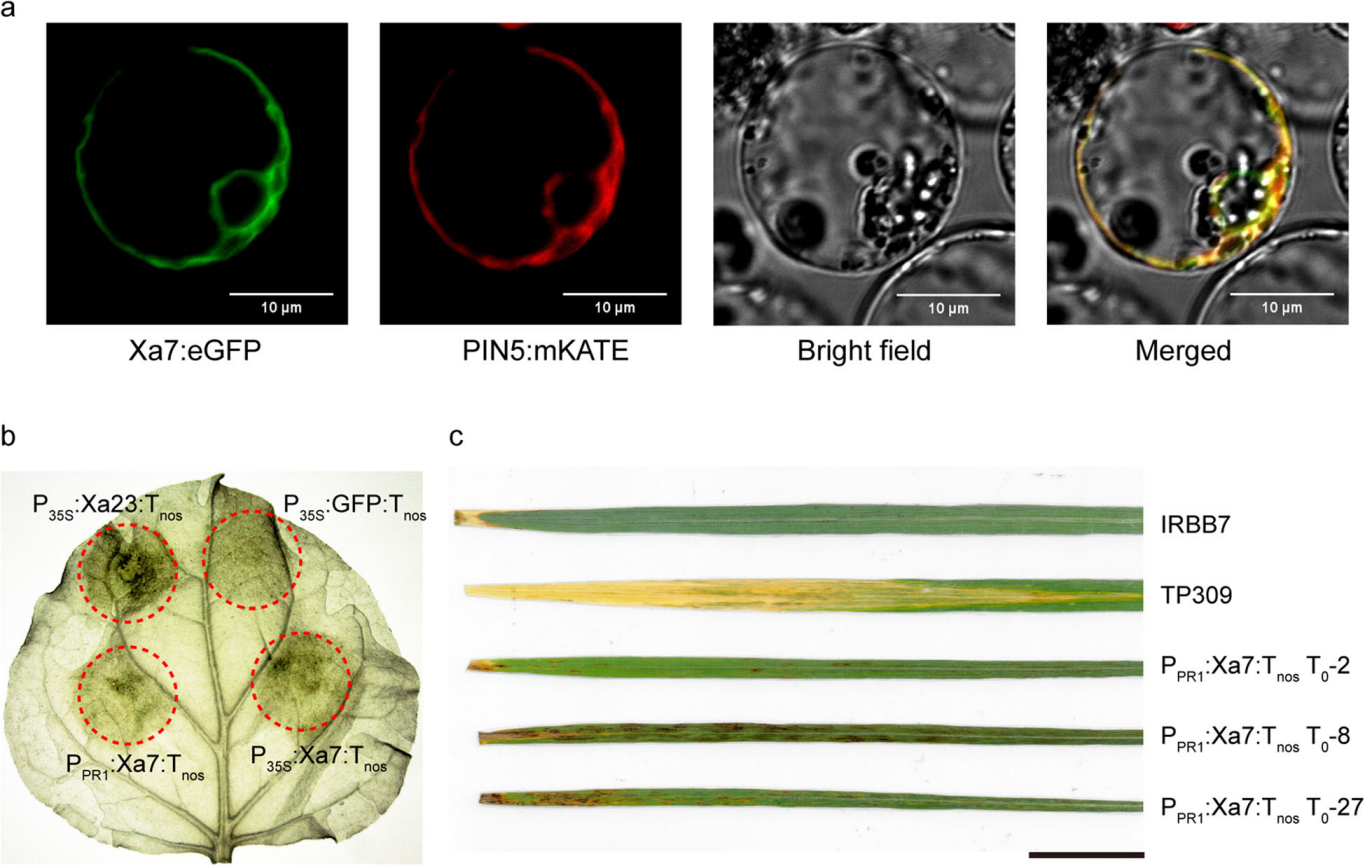
XA7 elicits a hypersensitive response in rice and tobacco (*Nicotiana benthamiana*). (**a**) XA7 is anchored to the endoplasmic reticulum (ER) membrane and co-located with the ER marker PIN5 in rice protoplasts. (**b**) *Xa7* expression induces cell death in the tobacco leaf following *Agrobacterium tumefaciens*–mediated infiltration. (**c**) *Xa7* causes spontaneous lesions on rice leaves generated stably expressiong a *P*_*PR1*_:*Xa7*:*T*_*nos*_ construction. Scale bar represents 5 cm

XA7 has been predicted to contain two transmembrane structures. The subcellular localization of protein is always adapted to its functional mechanism. XA7 was separately fused with an N-terminal eGFP or a C-terminal eGFP. A preliminary subcellular localization study using these constructs showed that both XA7:eGFP and eGFP:XA7 fusion proteins were localized to the nuclear envelope and the peripheral endoplasmic reticulum (ER) in the rice protoplasts (Fig. S3). Further experiments demonstrated that XA7:eGFP anchors to the ER and co-locats with the ER membrane marker PIN5:mKATE (Fig. 5a).

Executor resistance proteins typically limit pathogen expansion by triggering a localized cell death response. In this study, the mechanism of XA7 was verified using a tobacco transient expression system and a rice stable transformation system. The transient expression plasmids were constructed by fusing the *Xa7* CDS with the constitutive promoter *35S* or pathogen inducible promoter *PR1*, respectively. The fused constructs were transiently expressed in tobacco leaf cells following an *Agrobacterium tumefaciens*–mediated infiltration. Both the *P*_*35S*_:*Xa7*:*T*_*nos*_ and *P*_*PR1*_:*Xa7*:*T*_*nos*_ constructs elicited a cell death reaction in tobacco leaves (Fig. 5b). *P*_*PR1*_: *Xa7*: *T*_*nos*_ transgenic rice plants also developed spontaneous lesions in the leaves of positive lines generated from the stable genetic transformation (Fig. 5c). And *P*_*PR1*_:*Xa7*:*T*_*nos*_ transgenic rice also exhibited resistance to PXO86 (Table 1). According to the similar mode of action to other reported executor genes, including *Bs3*/*Bs3-E* (Römer et al., 2007), *Bs4C-R* (Strauß et al., 2012), *Xa27* (Gu et al., 2005), *Xa10* (Tian et al., 2014), and *Xa23* (Wang et al., 2015), *Xa7* can be classified as the same group of *R* genes.

### The Alleles of *Xa7* Are Present in the Outer Branch of the Phylogenetic Tree within the *Oryza* Genus and its Outgroup *Leersia perrieri*

**Fig. 6:**
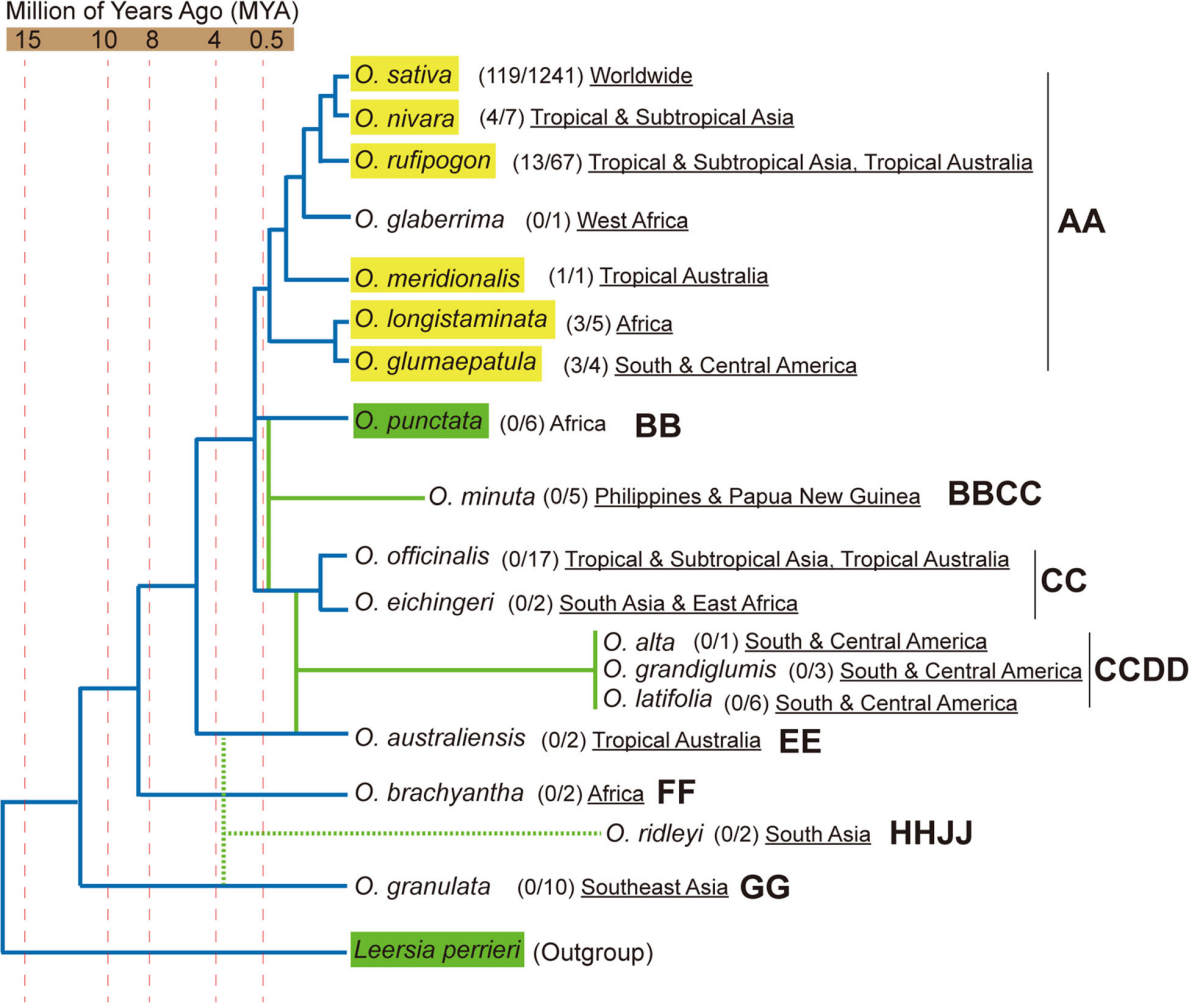
*Xa7* alleles survey in the *Oryza* genus. The phylogenetic tree of the *Oryza* genus and outgroup *Leersia perrieri* was modified from the articles by Kellogg (2009) and Jacquemin et al. (2013). The divergence time is based on data from the literature (Ammiraju et al. 2008; Guo and Ge, 2005; Tang et al. 2010; Wang et al. 2009). The result of the *Xa7* CDS amplification is shown in brackets: The denominators indicate the number of landraces and wild rice species used for the detection, while the numerator indicates the positive number of the detections. Geographical distribution information is underlined. The genome type is indicated in bold font. The species carrying *Xa7* alleles based on experimental detection are highlighted in yellow. The species carrying *Xa*7 homologs based on a BLAST at UniProt database, are highlighted in green. Accessions with a hit Score > 300, an identity > 60%, and an E-value <1e-30 were classified as XA7 homologs

**Fig. 7:**
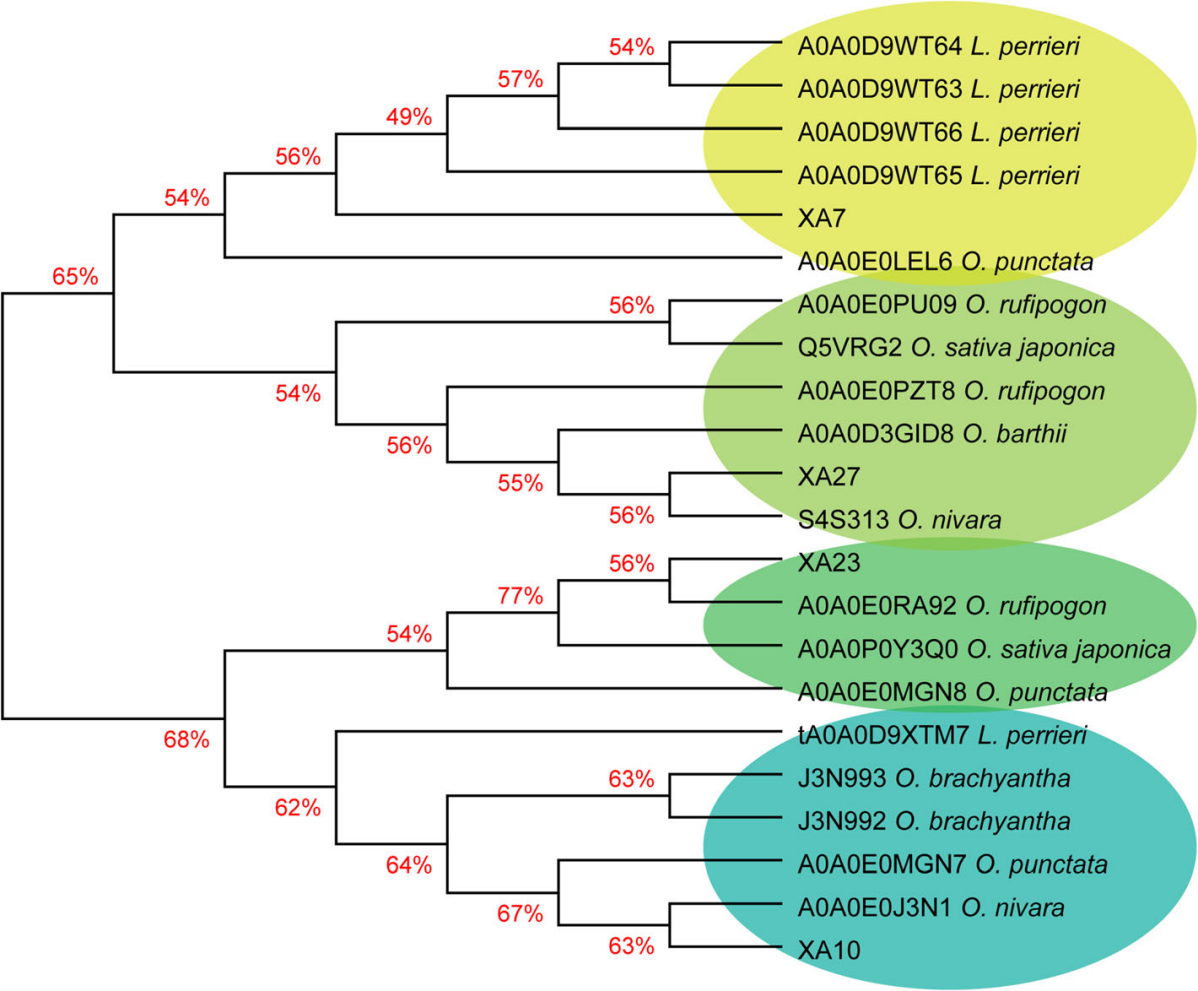
Phylogenetic analysis of XA7, XA10, XA23, XA27, and their homologs. Five homologous proteins with the highest hit scores for the rice executor proteins were downloaded from the UniProt protein resource database, separately. Sequence alignment was performed using ClustalW method. The tree was constructed using the neighbor-joining method

*Xa7* is absent in the reference genomes, including the *japonica* cultivar Nipponbare, and the *indica* cultivars Minghui63, Zhenshan97, and 93–11. To survey the distribution of the *Xa7* locus in the *Oryza* genus, we had analyzed representative rice germplasm collected from different geographic regions, including 1241 cultivars and landraces from the 3 k RGP and 141 accessions of wild rice (Fig. 6). We detected the presence of *Xa7* by performing PCR using *Xa7* CDS–specific primers. Only 9.6% of cultivars and landraces were found to carry the *Xa7* locus, most of which belong to the *aus/boro* and *indica* subspecies of rice from India, Bangladesh, and other countries in South Asian. *Xa7* alleles were detected in 17% of the wild rice species investigated, all of which belong to the AA genome type. These wild rice lineages are widely distributed across four continents, including East and South Asia, Africa, South and Central America, and Oceania. The wild rice lineages carrying the *Xa7* locus were positioned in the outer branches of the phylogenetic tree within the *Oryza* genus, while the lineages lacking the *Xa7* locus, such as *O. granulata*, *O*. *brachyantha* and *O. australiensis*, were close to the root of the phylogenetic tree (Fig. 6). In the UniProt protein database (https://www.uniprot.org/blast/), the proteins with the highest homology score for XA7 were from the grass *Leersia perrieri*, which was the nearest outgroup of the *Oryza* genus, while the homologs with the highest score for other rice executor R proteins, including XA10, XA23, and XA27, were almost all from the same genus of *Oryza* (Fig. 7). Moreover, the matched homologs from *Leersia perrieri* had undergone a gene duplication event. There were five members of the gene family distributed in a cluster on chromosome 6 of *Leersia perrieri* (Fig. S4), and their sequence identity with XA7 ranged from 55.93% to 66.37% (Fig. S2).

## Discussion

*Xa7* is known to be a durable resistance gene. The mechanism of its durability can be attributed, in part, to two factors. First, the resistance level of *Xa7* is dynamic. Previous studies showed that the resistance level yielded by *Xa7* could be enhanced by high temperature, drought, and other abiotic stresses (Webb et al. 2010; Dossa et al., 2020). The dynamic level of resistance does not maintain persistent and excessive selection pressure on pathogens, slowing down the rate at which they overcome *Xa7*. In long-term field performance, the resistance of rice cultivars carrying *Xa7* could not be easily broken down. Another reason for durable resistance of *Xa7* in the field can be attributed to its cognate avirulence gene *AvrXa7*, which can also activate the susceptible rice gene *SWEET14*. *SWEET14* encodes a sugar transporter and elevated expression of *SWEET14* is conducive to the proliferation of *Xoo* in rice. According to previous studies, the EBE_AvrXa7_ of *SWEET14* exists in a high proportion of 3000 landraces and cultivars (Zaka et al. 2018), however, our study shows that *Xa7* is present only in a small proportion of 3000 rice accessions. From the perspective of the pathogen population, the preservation of AvrXa7 in the genome is more beneficial. *SWEET14* and *Xa7* are just like a carrot and a stick for the pathogen, therefore, *Xoo* prefers to retain *AvrXa7* to promote *SWEET14* activity, despite resulting in a fitness penalty for the *Xa7* gene. As a result, varieties harboring *Xa7* would show more durable resistance in the field.

It took almost two decades for multiple groups to molecularly clone *Xa7* because of a large genomic structural variation (SV) present in this gene mapping region (Chen et al. 2021; Luo et al. 2021, two papers published during the preparation of this manuscript). Many types of SVs exist in the genome, such as deletions, duplications, copy-number variants, insertions, inversions, and translocations. SVs typically affects a sequence length from about 1 kb to 3 Mb, which is larger than a single-nucleotide polymorphism, but smaller than a chromosome abnormality. The analysis of these SV regions may enable the identification of new *R* genes, as *R* genes are often present in SV regions. The reason for this is that R genes are constantly under strong selection pressure; thus, their origination and loss from the genome are rapid. A pan-genome analysis revealed that the genes of SV regions were enriched in abiotic and biotic stress response genes, especially for NBS–LRR and B–ARC (a nucleotide-binding adaptor shared by APAF-1, R proteins and CED-4) genes, which control disease resistance in rice (Zhao et al. 2018). SV information is generally missing or not assembled correctly when using the next-generation genome sequencing technology. With the widespread use of the third-generation sequencing platforms and the decrease in cost, more and more SV information will be uncovered and potentially used to mine for functional disease resistance genes.

In addition to our work, two recently published papers also identified the gene encoding a 113-amino-acid protein and validated its function as *Xa7* (Chen et al. 2021; Luo et al. 2021). The three independent research groups adopted different research strategies to identify the functional *Xa7* gene from the large SV region. Chen et al. constructed a large radiation-mutagenesis library of Zhen-hui 084 (the variety containing *Xa7*), and screened for susceptible mutant lines. They compared the SV sequence of the mutants with Zhen-hui 084 to narrow down the locus to a 28-kb region and finally isolated the functional *Xa7* gene. Luo et al. used CRISPR/Cas9-mediated site-directed mutagenesis to produce large deletions, which further localized *Xa7* to a 53-kb region. They performed a RAMPAGE analysis, which combines RNA annotation and mapping of the respective promoters, discovering the 113-amino acid small protein induced by AvrXa7. Our group developed an alternative approach to identify the candidate gene using the web-based tool TALgetter to predict AvrXa7-targeted genes in the 106-kb genomic gap sequence. Two AvrXa7 EBEs were identified with a *p*-value less than 1.0 × 10^− 6^. One of them was located in the intron of a transposase gene; therefore, we excluded this EBE. The other EBE was located in the promoter region of the 113 aa ORF and this was considered as the candidate *Xa7* gene. This knowledge-based strategy saved much time compared with traditional approaches.

Wild rice species in the *Oryza* genus are an important resource for mining genes that confer disease resistance and other agronomic traits. Many important resistance genes were identified from wild rice, including *Xa21* from *O. longistaminata* (Song et al. 1995), *Xa27* from *O. minuta* (Gu et al. 2005), *Xa23* from *O. rufipogon* (Wang et al. 2015), *Pi9* from *O. minuta* (Qu et al. 2006), *Bph10* from *O. australiensis* (Ishii et al., 1994), and *Bph14* from *O. officinalis* (Du et al. 2009). The homologs of these genes could be traced back to the common lineages of the *Oryza* genus. By contrast, *Xa7* is absent in most wild rice lineages, but homologous alleles could be found in the *Leersia* genus. *Oryza* and *Leersia* belong to the same tribe, the *Oryzeae*, and are closely related members of the *Poaceae*. *Leersia* species are often used as an outgroup of the *Oryza* genus in phylogenetic and genomic investigations (Copetti et al. 2015). Evidence of genome duplication events shared by *Oryza* and *Leersia* has been reported (Jacquemin et al. 2009). The phytocassane biosynthetic gene cluster in cultivated rice (*Oryza sativa*) is present in *Leersia perrieri* (Miyamoto et al., 2016). Genetic variation at the *Sub1* locus is due to gene duplication and divergence that occurred both prior to and after rice domestication. The *SUB1A-like* genes were also found in *Leersia perrieri*, which is tolerant to deep-flooding (dos Santos et al. 2017). *OsDR10*, a negative regulator of rice disease resistance against *Xoo*, is an intronless gene and its homologs exist in *Oryza* and *Leersia* (Xiao et al. 2009). *Leersia* plants are also threatened by *Xanthomonas* sp. As early as 1957, Chinese pathologists first reported that *Xanthomonas campestris* pv. *leersiae* (*Xcl*) is pathogenic to *Leersia hexandra*, producing water-soaking streaks with bacterial exudate on leaves. *Xcl* also showed weak pathogenesis in rice following artificial inoculations (Fang et al. 1957). A recent study identified the type-III effector repertoires (TALomes) in the *Xcl* genome and provided evidence to support the renaming of *Xcl* to *X. oryzae* pv. *leersiae* (*Xol*) (Lang et al. 2019). Potential *Xol* TALE gene targets have also been predicted in draft *Leersia* genome sequences, therefore, the *Oryza* and *Leersia* genera probably share similar mechanisms underlying the interaction between hosts and pathogens. The previous and present studies indicate that the discovery of new resistance genes need not be limited to wild rice, but could instead be extended to wild grass such as members of the *Leersia* genus.

The *Xa7* alleles are present in a small proportion of cultivars, landraces and wild rice and show regional preference, indicating that they are under strong selection pressure and would only be retained in specific ecological environments. *Xa7* alleles were not detected in the primitive lineages (EE, FF, and GG genome types) near the base of the phylogenetic tree in *Oryza*, but are present in the newly evolved lineages (AA and BB genome types) at the distal branch of the phylogenetic tree (Guo and Ge, 2006). It is conceivable to presume that *Xa7* might initially exist in the common ancestor of *Oryza* and *Leersia*, with gradual elimination events occuring in the root lineages of *Oryza* during the evolutionary process. Alternatively, an intergeneric hybridization event may be occurred between *Leersia* and the outer branch lineages of the *Oryza*. The loss event or distant hybridization event of the *Xa7* locus likely occurred after the split of the root lineages (EE, FF, and GG genome types) from the outer branch lineages (AA and BB genome types), and before the divergence of the AA and BB genome species. The estimated time this occurred is between 4 MYA and 0.5 MYA. The proposed events in both hypotheses occurred well after the Gondwanaland fragmentation and drift (at least 100 MYA), therefore, the global geographical distribution pattern of the *Xa7* alleles might be the results of transoceanic dispersal or long-distance dispersal.

## Conclusions

The bacterial blight resistance gene *Xa7* in rice was functionally confirmed using map-based cloning. The *Xa7* locus in the line IRBB7 is located in an approximately 100-kb region that is non-collinear with the rice Nipponbare reference genome. A small gene encoding a unique 113-amino-acid protein was validated for *Xa7* function. The transcription of *Xa7* is induced by AvrXa7. The AvrXa7 binding element is located in the *Xa7* promoter region, which acts as a trap to perceive the avirulent effector. XA7 is anchored in the endoplasmic reticulum membrane and its ectopic expression triggers cell death in monocot and dicot plants. An allele analysis provides clues to the evolutionary origin of *Xa7*. Functional identification of the *Xa7* gene will facilitate further studies to reveal the mechanism of durable and stress-enhanced resistance, which would extend to many broad applications.

## Materials and Methods

### Plant Materials and Bacterial Inoculation

IRBB7 is the rice near-isogenic line of *Xa7* gene in the IR24 genetic background (Ogawa et al. 1991). II-32B, IR24 and Zhonghua11 (ZH11) are *indica* varieties susceptible to *Xoo* PXO86. TP309 is a susceptible *japonica* variety. The mapping population was generated from the cross between II-32B and IRBB7, and was grown in the field. Transgenic plants were grown in the greenhouse at 32 °C for 14 h (light) and 28 °C for 10 h (dark). Dr. Jianlong Xu (Institute of Crop Sciences, Chinese Academy of Agricultural Sciences) kindly provided 3000 rice landraces. Leaves of wild rice accessions were collected from the national germplasm Guangzhou wild rice nursery.

*Xoo* strains were cultured in NA_0_ medium at 28 °C. Bacterial suspensions were adjusted to an optical density of 0.5 at 600 nm, then used to inoculate rice plants at the booting (panicle development) stage using the leaf tip–clipping method. The disease level was evaluated 2 weeks after inoculation by measuring the lesion length as described previously (Chen et al. 2008).

### IRBB7 BAC Library and TAC Sub-Library Construction

The CopyControl™ pCC1BAC™ (Epicentre, USA) vector was used to construct the IRBB7 BAC library. The pYLTAC747H/sacB (Liu et al. 1999; Liu et al. 2002) vector was used for the construction of the sub-libraries and the rice transformation. Genomic DNA was extracted from etiolated seedlings of IRBB7. The DNA purification, partial digestion with *Hind*III, pulsed field gel electrophoresis, ligation and library construction were carried out as described previously (Liu and Whittier, 1994). The flanking markers U05 and Poz (Table S1) were used for the library screening. The BAC plasmids P1-10G and P3-12F carrying candidate genes were partially digested with *BamH*I and *Sau3A*I, respectively. DNA fragments of 8 ~ 13-kb in length were collected for the construction of sub-libraries.

### BAC Plasmid Sequencing and Assembly

BAC plasmids were isolated using a BAC/PAC DNA Isolation Kit (OMEGA). The extracted DNA was subjected to quality control by agarose gel electrophoresis and quantified by Qubit. The BACs were sequenced with massively parallel sequencing Illumina technology. Library construction and sequencing was performed at the Beijing Novogene Bioinformatics Technology Co., Ltd. Two DNA libraries were constructed: a paired-end library with an insert size of 500 bp and a mate-pair library with an insert size of 5 kb. These libraries were sequenced using an Illumina HiSeq2500 platform following PE125 strategy. Illumina PCR adapter reads and low-quality reads from the paired-end and mate-pair libraries were filtered by the step of quality control using our own compiling pipeline. All good quality paired reads were assembled using the SOAPdenovo 2.04 (http://soap.genomics.org.cn/soapdenovo.html) into a number of scaffolds. Then the filtered reads were then handled by the gap-closing process.

### Plasmid Construction and Rice Transformation

The single *Xa7* candidate gene, including the 458-bp promoter and full CDS sequence, was amplified from the genomic DNA of IRBB7 using KOD-Plus-Neo (TOYOBO, Japan) with the recommended method. The resultant PCR products were ligated into the pCambia1300Asc1 expression vector. The complementary construct and selected TAC sub-clone plasmids were delivered separately into the *Agrobacterium tumefaciens* strain EHA105. The rice transformation was then performed using the rice callus of the *indica* varieties IR24, ZH11, or the *japonica* variety TP309 by the *Agrobacterium*-mediated method. T_0_ or T_1_ generation transgenic lines were inoculated with PXO86 at the booting stage to identify their resistance phenotypes.

### Full-Length cDNA Determination

Total RNA was isolated from the leaf tissues using NucleoZOL (Macherey-Nagel, Germany) following the manufacturer’s instruction. First-strand cDNA was synthesized using a SMART RACE cDNA Amplification Kit (Clontech, Japan). The 5′ UTR and 3′ UTR regions were PCR-amplified by following the standard protocol. Gene-specific primers are provided in Table S1. All PCR products were gel-purified, cloned (pEASY™-Blunt Zero Cloning Kit, TRANS), and sequenced.

### Relative Quantification of Gene Expression

The 5-cm leaf sections next to the bacterial infection sites were harvested for RNA isolation. cDNA was synthesized using a PrimeScript™ RT reagent Kit with gDNA Eraser (Takara, Japan). A quantitative-PCR was performed using SYBR® Premix Ex Taq™ II (Tli RNaseH Plus) (Takara, Japan) on a CFX96™ platform (Bio-Rad Laboratories). The rice *TF2* gene was served as an endogenous control. The *TF2* and *Xa7* gene-specific primers are shown in Table S1. All reactions were run in triplicate. The average threshold cycle (Ct) was used to determine the fold change in gene expression. The 2^-△△Ct^ method was used for the relative expression quantification, and the results are presented as the mean ± standard deviation.

### CRISPR/Cas9-Mediated Site-Specific Gene Knockout

Binary CRISPR/Cas9 vector (pYLCRISPR/Cas9P_ubi_-H) and sgRNA intermediate vectors (pYLsgRNA-OsU6aL, pYLsgRNA-OsU3aL, and pYLsgRNA-OsU6c) were kindly provided by Dr. Yaoguang Liu (College of Life Sciences, South China Agricultural University). The selection and design of sgRNA target sequences were based on the web-tool CRISPR-P (http://crispr.hzau.edu.cn/CRISPR/) (Lei et al. 2014). Firstly, the PAM sequences were searched by CRISPR-P within the EBE_AvrXa7_ and *Xa7* CDS region. Appropriate target sites were selected and corresponding sgRNA adaptors were designed (the adaptor sequences are listed in Table S1). The site-specific editing vectors were constructed following the standard operating procedure (Ma et al. 2015; Ma and Liu, 2016): the target adaptor was ligated with *Bsa*I-digested intermediate vector, and after two rounds of PCR, annealing and nested PCR amplification, the specific sgRNA expression cassette was obtained. The cassette was then assembled into a binary expression vector. The target1 and target2 editing vector was constructed individually, while target3 and target4 were constructed into the same binary vector. The three binary vectors were transferred separately into IRBB7 using *Agrobacterium*-mediated gene delivery. All transgenic lines were genotyped using PCR and direct Sanger sequencing of the PCR-amplicons containing the targeted sites. The sequencing chromatograms were decoded by DSDecode (Liu et al. 2015). Homozygous T_1_ or T_2_ mutants were selected for the following disease resistance evaluation (described above).

### Subcellular Localization of XA7

The *Xa7* CDS fragment was separately fused with the N-terminus of eGFP or the C-terminus of eGFP. The transient expression of the XA7:eGFP, eGFP:XA7 and eGFP constructs was driven by the 35S promoter in rice protoplasts mediated by polyethylene glycol. The resulting protoplasts were observed using a laser scanning confocal microscope at 16 h after inoculation. After a preliminary localization study, XA7:eGFP and PIN5:mKATE (ER membrane marker) constructs were co-transformed into rice protoplasts to validate the co-localization result.

### Hypersensitive Response Assays

Cell suspensions (OD_600_ = 1.0) of *A. tumefaciens* strains harboring *P*_*35S*_:*Xa23*, *P*_*35S*_:*eGFP*, *P*_*35S*_:*Xa7* and *P*_*PR1*_:*Xa7* constructs were individually infiltrated into the leaves of *N. benthamiana* using needleless syringes. The leaves were sampled 4 days post-infiltration, then cleared in ethanol to visualize the symptom.

### Phylogenetic Analysis

The sequence of XA7 was used to query the UniProt protein resource database (https://www.uniprot.org/blast/) for homologous sequences using the BLAST program. The alignment and phylogenetic analysis of the sequences of the bacterial blight executor proteins and their close homologs was performed with DNAMAN (https://www.lynnon.com/dnaman.html/). The tree was constructed using the neighbor-joining method.

## Supplementary Information

**Additional file 1: Supplemental Figure 1.** Prediction of XA7 disorder using IUPred2 with default parameters.

**Additional file 2: Supplemental Figure 2.** Sequence alignment of XA7 with its homology proteins and reported executor proteins in rice.

**Additional file 3: Supplemental Figure 3.** Preliminary subcellular localization of XA7 in rice protoplasts.

**Additional file 4: Supplemental Figure 4.** Members of the *Xa*7 homologs gene family are distributed in a cluster on chromosome 6 of *Leersia perrier*.

**Additional file 5: Supplemental Table 1.** Primers used in this study.

## Data Availability

Sequence data from this article have been deposited in the GenBank: MW427595 for assembly of IRBB7 BAC clones (P2-9D, P3-12F, P1-10G) inserts sequence. All relevant data are provided within the article and its supplementary information files.

## Abbreviations

*Xoo*: *Xanthomonas oryzae* pv. *oryzae*
EBE: Effector binding element
EBE_AvrXa7_: Effector binding site of AvrXa7
*R* gene: Resistance gene
T3SS: Type-III secretion system
TALEs: T3SS transports transcription activator–like effectors
RVDs: Repeat-variable diresidues
3 k RGP: 3000 rice genome project
TAC: The transformation-competent artificial chromosome vector
ORFs: Open reading frames
ZH11: Zhonghua11
TP309: Taipei309
UTR: Untranslated region
TSS: Transcription initiation site
ATG: The initiation codon
ER: Endoplasmic reticulum
SV: Genomic structural variation
*Xol*: *Xanthomonas oryzae* pv. *leersiae*
