# Supplementary figures and images for: Xa7, a Small Orphan Gene Harboring Promoter Trap for AvrXa7, Leads to the Durable Resistance to Xanthomonas oryzae Pv. oryzae
Source: Rice (N Y). 2021 May 30;14:48. doi: 10.1186/s12284-021-00490-z (PMC8165051; doi:10.1186/s12284-021-00490-z)

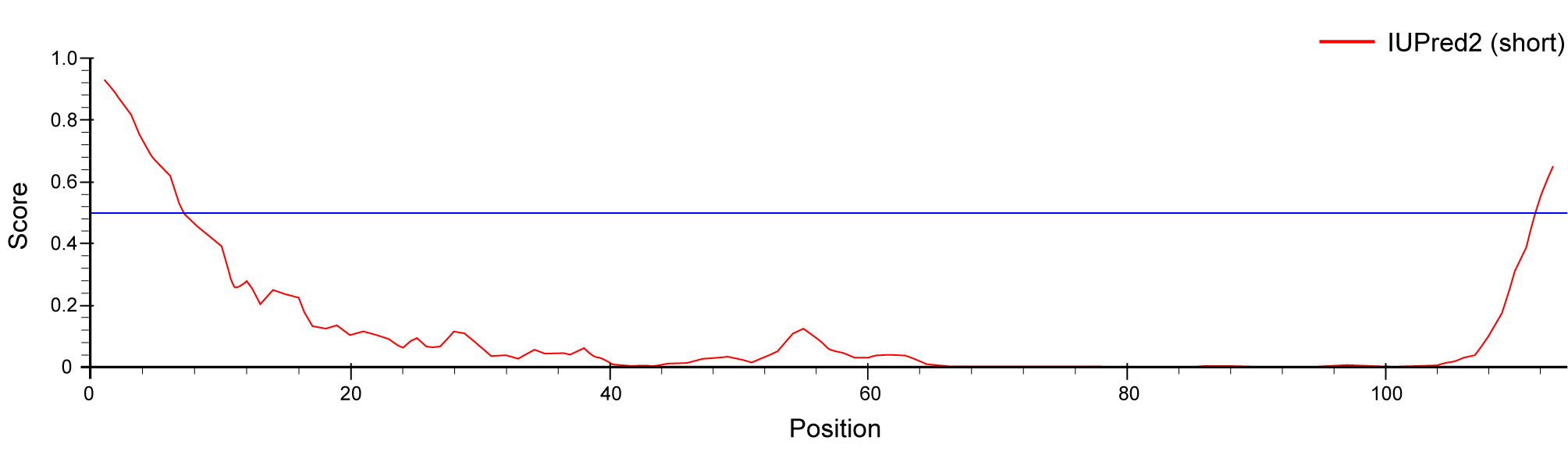

Supplement: Supplementary file 1 — Additional file 1: Supplemental Figure 1. Prediction of XA7 disorder using IUPred2 with default parameters. [file 12284_2021_490_MOESM1_ESM.tif]

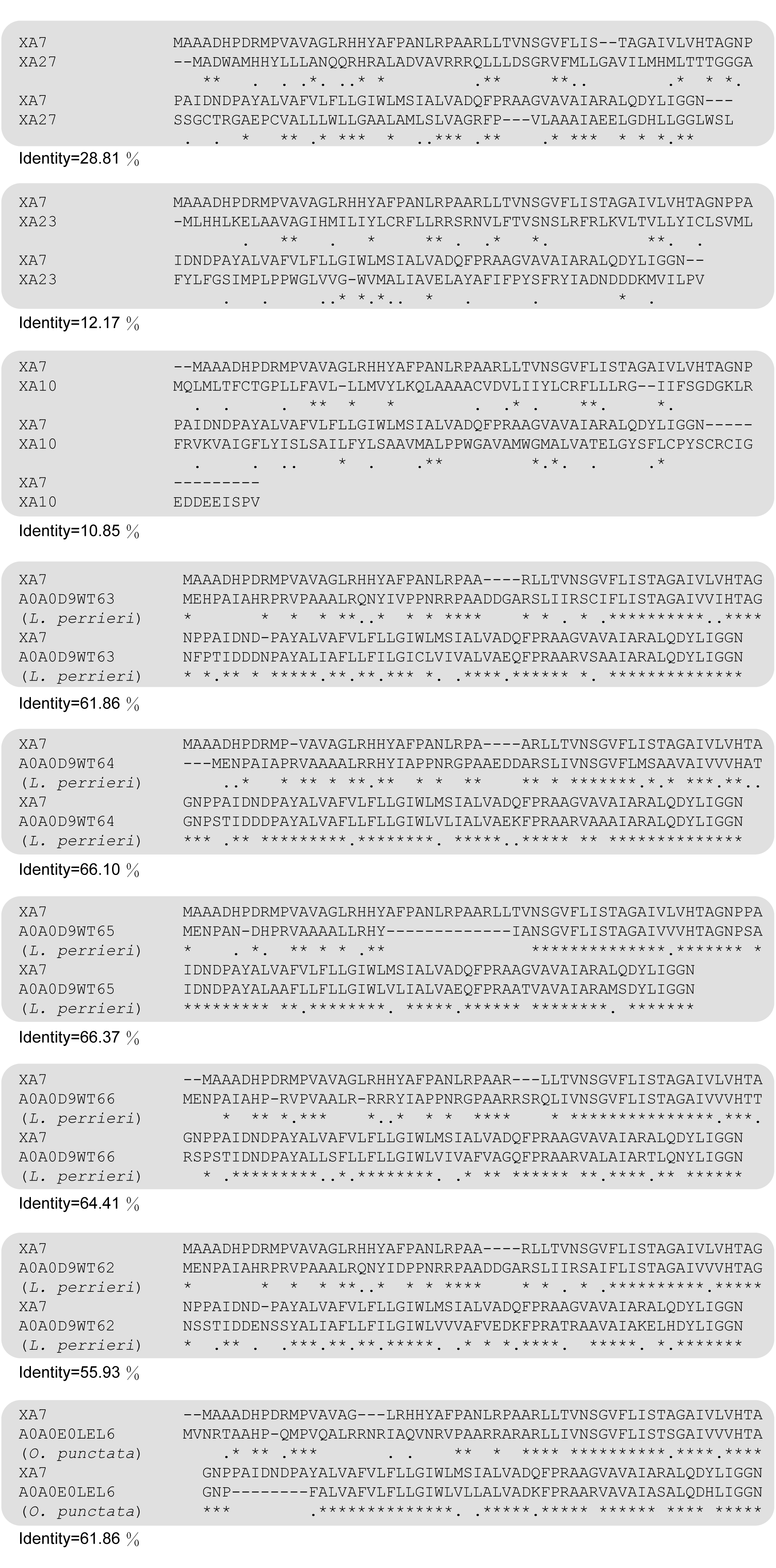

Supplement: Supplementary file 2 — Additional file 2: Supplemental Figure 2. Sequence alignment of XA7 with its homology proteins and reported executor proteins in rice. [file 12284_2021_490_MOESM2_ESM.tif]

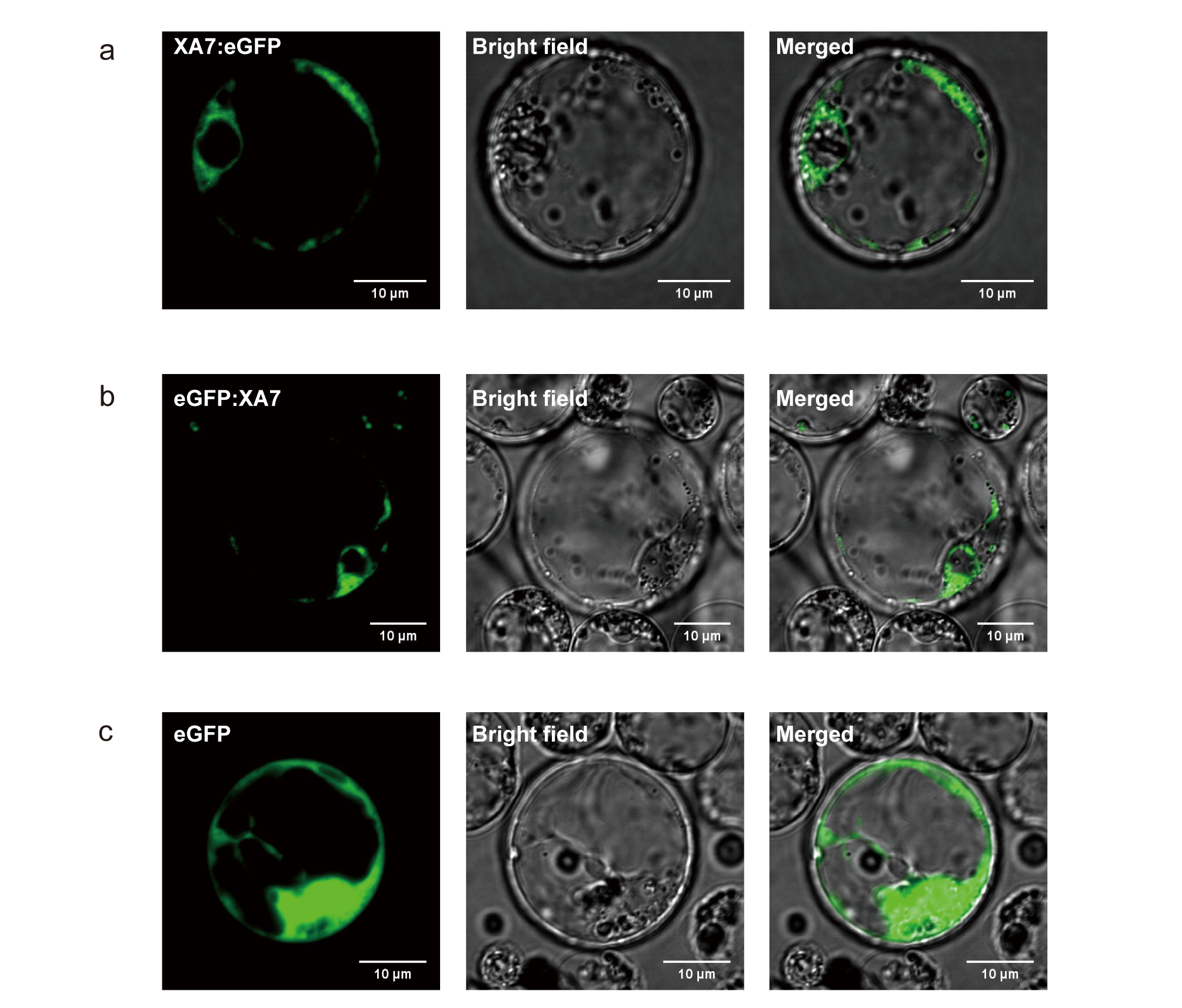

Supplement: Supplementary file 3 — Additional file 3: Supplemental Figure 3. Preliminary subcellular localization of XA7 in rice protoplasts. [file 12284_2021_490_MOESM3_ESM.tif]

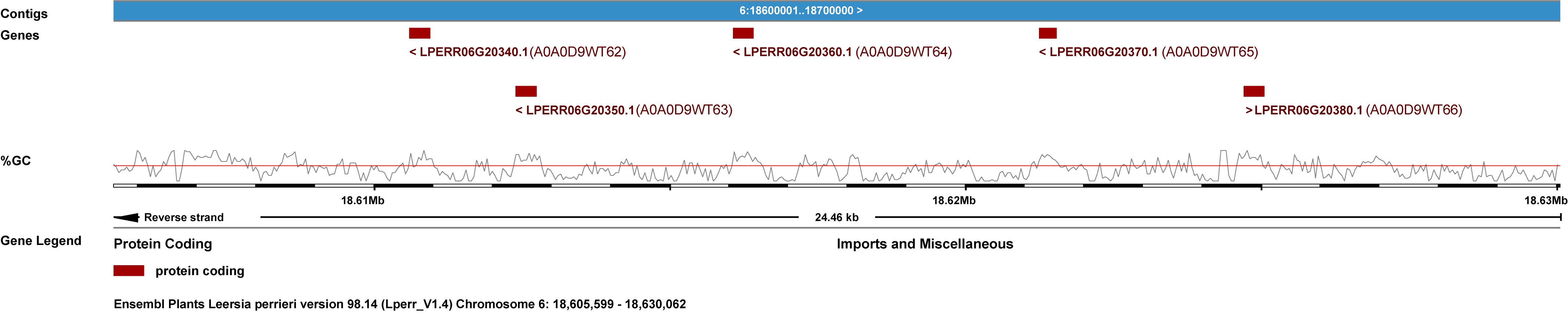

Supplement: Supplementary file 4 — Additional file 4: Supplemental Figure 4. Members of the Xa7 homologs gene family are distributed in a cluster on chromosome 6 of Leersia perrier. [file 12284_2021_490_MOESM4_ESM.tif]
