# Supplementary material for: Xa7, a Small Orphan Gene Harboring Promoter Trap for AvrXa7, Leads to the Durable Resistance to Xanthomonas oryzae Pv. oryzae
Source: Rice (N Y). 2021 May 30;14:48. doi: 10.1186/s12284-021-00490-z (PMC8165051; doi:10.1186/s12284-021-00490-z)
Supplement: Supplementary file 5 — Additional file 5: Supplemental Table 1. Primers used in this study. [file 12284_2021_490_MOESM5_ESM.doc]

**Supplemental Table**

**Supplemental Table1.** Primers used in this study.

| **Primer** | **Forward primer** | | **Reverse primer** | | **Usage** |
| --- | --- | --- | --- | --- | --- |
| **Fine mapping** |  | |  | |  |
| RM20582 | AGAGCGTCGTCCTTCACCATCC | | GGCCAATACGACGATACATTACACG | | Fine mapping |
| RM20593 | AAGGTACACTTGCTCTGACGGTAGC | | AGACCTCAGTGGCAAATCCTACG | | Fine mapping |
| U09 | CACATTGACTTGTCACGGCCATTG | | CGAGAAGTTCACAATCGTTAGGTC | | Fine mapping |
| U06 | GTGATGGTCTTTCCTGTCAAGGTC | | CTTCGCTGCGCGACCTAGTCAAC | | Fine mapping |
| U05 | CAGACAAGTGTTGTTCATGTTCG | | GAAGTCCGAGCTGGGGACGATGTAC | | Fine mapping and BAC library screening |
| U04 | GTTGACTTGACTTGATGTTATGCCCGTTTCAGAC | | GAAATGAGTTCCCGAGCCCGTGTAGTAGCTCTAG | | Fine mapping |
| U01 | GATATCTGGCGTCTCCTAACTAACC | | GAGAGAAGGTTGATGTAGCAGCC | | Fine mapping |
| Poz | ATGCAGCCCAAGAAAGGTCCAACTC | | GAGGGAGAGGACCTGGTCGGTGAG | | Fine mapping and BAC library screening |
| GDSSR02 | TGCCCACCGTCGATCTCGTGG | | AGCTAGCAATTCGCATGATTGC | | Fine mapping |
| 71SR | TACACCGCCGTCACCACTCTGAC | | TCTCTCCCCTTCGGACGCAT | | Fine mapping |
| RM20591 | TCGTCTGCGCGAATATTTAGAGAGG | | ATCTGCATCGGAGTCAGCAACG | | Fine mapping |
| **BAC library screening and BAC-end sequencing** | | | | | |
| pCC1 BAC end | GGATGTGCTGCAAGGCGATTAAGTTGG | | CTCGTATGTTGTGTGGAATTGTGAGC | | BAC-end sequencing primer |
| pYLTAC747H end | TGACATTGTAGGACTATATTGCTC | | TCATGTCTCCTTCTGTATGTACTG | | Subclone-end sequencing primer |
| Xa7 CDS | GATCGTATGCCCGTTGCAGTTGC | | TGCCACCGATGAGGTAATCCTGC | | Sub clones screening |
| Xa7 promoter | GCAAAAGATGTATGGGAGCATACC | | GGATGATGGATCCCCCAGGTTG | | Sub clones screening |
| Xa7-1300Asc1 Vet | CTATGGCGCGCCTCAGTTCTACCGGGGTTCAGTTC | | CTATGGCGCGCCATGAAGCAGAAAATACGAC | | Vector constructed for complementary transformation |
| **Complementary transgenic lines validation** | | | | | |
| HPT | GGTTTCCACTATCGGCGAGTACTTC | | CGTGCTTTCAGCTTCGATGTAGG | | Hygromycin resistance gene detection |
| Xa7-1300 DT | CCTCCTCGGAATCTGGCTCATGTC | | GCAAGGCGATTAAGTTGGGTAACG | | Specific primer for the detection of the Xa7-1300Asc1 construct |
| S1AE6 | TCATGTCTCCTTCTGTATGTACTG | | GCAATGTTCGTTAGCGGGAGG | | Specific primer for S1AE6 subclone detection |
| S1AG3 | TCATGTCTCCTTCTGTATGTACTG | | CTCAACTAGGAGCAAACATACACC | | Specific primer for S1AG3 subclone detection |
| S2CD8 | GAAATTTCACAGAGGAGGGACTGG | | CCATGAAGCAGAAAATACGAC | | Specific primer for S2CD8 subclone detection |
| S1BA3 | TGACATTGTAGGACTATATTGCTC | | GATCGTATGCCCGTTGCAGTTGC | | Specific primer for S1BA3 subclone detection |
| S2BD2 | TCATGTCTCCTTCTGTATGTACTG | | GGAGTTGACGGTCAGCAGTCGAG | | Specific primer for S2BD2 subclone detection |
| S1AD4 | TGACATTGTAGGACTATATTGCTC | | TGCCACCGATGAGGTAATCCTGC | | Specific primer for S1AD4 subclone detection |
| **qRT-PCR primers** | | | | | |
| *Xa7* qRT-PCR | GATCGTATGCCCGTTGCAGTTGC | | GGAGTTGACGGTCAGCAGTCGAG | | qRT-PCR for *Xa7* |
| TF2 | GCCTGAAGTGTACTGTACCACCAC | | CAAAGGGTTCAGAAATGAGGAA | | qRT-PCR endogenous control |
| **RACE primers** |  | |  | |  |
| *Xa7* 5'RACE |  | | TGCCACCGATGAGGTAATCCTGC | | *Xa7* 5'RACE specific primer |
| *Xa7* 3'RACE | CCTCCTCGGAATCTGGCTCATGTC | |  | | *Xa7* 3'RACE specific primer |
| **CRISPR/Cas9-mediated mutation** | | | | | |
| OsU6aT1 | gccgTATGTGGTTATCTGGGGGGG | | aaacCCCCCCCAGATAACCACATA | | *Xa7* promoter mutation Target1 |
| OsU6aT2 | gccgTTCGTATGTGGTTATCTGG | | aaacCCAGATAACCACATACGAA | | *Xa7* promoter mutation Target2 |
| OsU3T3 | ggcaCTGCAACGGGCATACGATC | | aaacGATCGTATGCCCGTTGCAG | | *Xa7* CDS mutation Target3 |
| OsU6cT4 | tcagCGACTGCTGACCGTCAACTC | | aaacGAGTTGACGGTCAGCAGTCG | | *Xa7* CDS mutation Target4 |
| Mutant DT | GAACTGCTCTGCTCAAGTGCCTC | | TGCCACCGATGAGGTAATCCTGC | | Mutants detection |
| **Subcellular localization** | |  | |  | |
| CG52Nosgfp | gggggactttgaggcaacATGGCGGCCGCTGATCATCCTGATC | | ctgaagcggccgctgtacAATTGCCACCGATGAGGTAATCC | | XA7-N'terminor-OsGFP vector construction |
| CG52Cosgfp | gacgagctctacaacatgGCGGCCGCTGATCATCCTGATC | | gaagacagagctagttacaTTAATTGCCACCGATGAGGTAA | | XA7-C'terminor-OsGFP vector construction |
| **Construction of over-expression and pathogen-induced expression vector** | | | | | |
| 35S Xa23 | gggactttgaggcaacATGTTGCATCATCTCAAGGAGC | | gacagagctagttacaTTAAACAGGGAGAATAACCATC | | Over-expression Xa23 vector construction |
| 35S Xa7 | gggactttgaggcaacATGGCGGCCGCTGATCATCCTGATC | | gacagagctagttacaTTAATTGCCACCGATGAGGTAA | | Over-expression Xa7 vector construction |
| CO39 PR1 promoter | GCCACATGACGGCTTGAGAGTG | | CAATGGCCAGCTTGGATACCTCC | | Pathogen-induced expression vector construction |
